# Case Report: Post-hip tuberculosis surgery: a rare case of giant iliac intraosseous epidermoid cyst

**DOI:** 10.3389/fonc.2025.1534753

**Published:** 2025-09-25

**Authors:** Guo Wei, Jie Liang

**Affiliations:** Department of Orthopedics, The First College of Clinical Medical Science, China Three Gorges University; Yichang Central People’s Hospital, Hubei Provincial Clinical Research Center for Osteoporotic Fractures, Yichang, Hubei, China

**Keywords:** intraosseous epidermoid cyst, ilium, hip joint tuberculosis, radiographs, MRI

## Abstract

**Background:**

Intraosseous epidermoid cyst (IEC) is a rare, non-neoplastic benign lesion. Acquired IECs are extremely rare and are predominantly attributed to trauma or iatrogenic interventions. The present case pertains to an iliac bone epidermoid cyst that emerged subsequent to surgery for hip joint tuberculosis, and to date, no corresponding documentation has been recorded in the extant literature.

**Case summary:**

A 43-year-old female, who had undergone an operative intervention for right hip joint tuberculosis 35 years ago, presented with a one-year history of activity-induced pain in the right hip. The radiographs revealed a narrowed joint space of the right hip joint, a shortened femoral neck, ischemic necrosis of the femoral head, and a large expansile radiolucent lesion with cortical erosion in the right iliac bone. Computed tomography (CT) demonstrated significant osteolytic destruction in the right iliac wing, along with a mass in the surrounding tissue. The patient underwent biopsy confirming the diagnosis of an IEC. She was successfully treated with curettage and allogeneic bone grafting.

**Conclusion:**

Despite the extreme rarity of this event, we should be aware of the potential *de novo* development of epidermoid cyst in patients who underwent surgery in the pelvic region, especially those related to surgery for hip joint tuberculosis. Once the diagnosis is made, thorough intra-lesional curettage with bone graft can achieve better postoperative outcomes.

## Introduction

Intraosseous epidermoid cyst (IEC) is an extremely rare benign mass of epithelial cell origin and presents as a lytic lesion or a pseudotumor. It usually occurs on the distal phalanges of the fingers and the skull (1–3). This phenomenon results from the unique local anatomy, since the subungual bone bed is attached to the underlying periosteum. Occasionally, involvement of the radius, tibia, femur, sacral and mandible can also be encountered (4–8). The origin of epidermoid cysts is still controversial. Although they are generally regarded as congenital, acquired origins have also been reported. Acquired IECs are extremely rare and are predominantly attributed to trauma or iatrogenic interventions such as lumbar puncture or surgical procedures. To the best of our knowledge, this is the first report concerning the formation of IEC of the ilium after surgery for hip joint tuberculosis. We will present this case and review the literature for known causes of IECs, physiopathology, radiologic features and management strategy of this rare pathology.

## Case description

A 43-year-old woman presented with limping and activity-related right hip pain. The patient reported that the pain had an insidious onset and progressed gradually over the last year, and described it as being of mild-to-moderate intensity. The pain was exacerbated with movement, walking, sitting down, and getting up, with no significant diurnal variation. Notably, there was a history of operative intervention for tuberculosis of the right hip joint at the age of eight. Unfortunately, we possess no documents (such as imaging data or operation reports) related to this surgery. The patient reported relief of hip joint pain, yet there are sequelae including limited hip joint mobility and shortening of the right lower limb after the operation. There was an aged surgical scar, approximately 20 cm long, which could be clearly seen on the exterior of the right hip joint. The right lower limb was approximately 5 cm shorter than the contralateral limb.

Physical examination revealed that a mass with a transverse diameter of about 10 cm could be felt at the anterior superior iliac spine of the right ilium. It demonstrated the range of motion of the hip as follows: 10° of abduction, 10° of adduction, 30° of flexion, 5° of buckling and 5° for both external rotation and internal rotation. The Trendelenburg’s test was positive on the affected side. The gait examination showed obvious abductor lurch. Other joints were normal.

**Figure 1:**
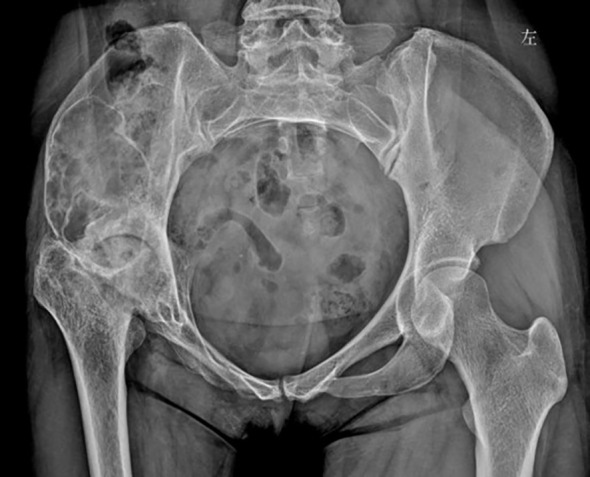
The pelvic plain film showed a narrowed joint space of the right hip joint, a shortened femoral neck, and ischemic necrosis of the femoral head. Also, radiographs revealed a large expansile radiolucent lesion with cortical erosion in the right iliac bone. 左: left.

**Figure 2:**
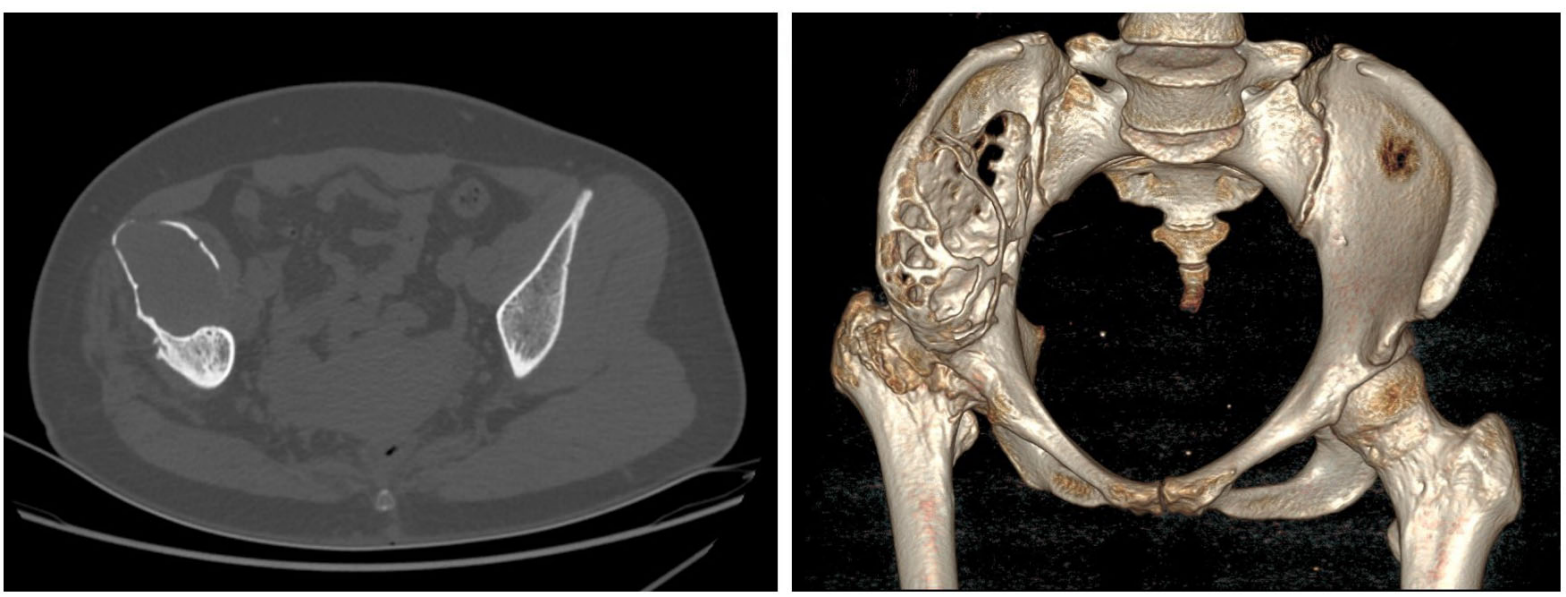
The CT scan demonstrated right ilium bone destruction, a surrounding ~10.3×6.2×7.8 cm soft tissue mass with irregular cysts, and an unclear muscle tissue boundary.

**Figure 3:**
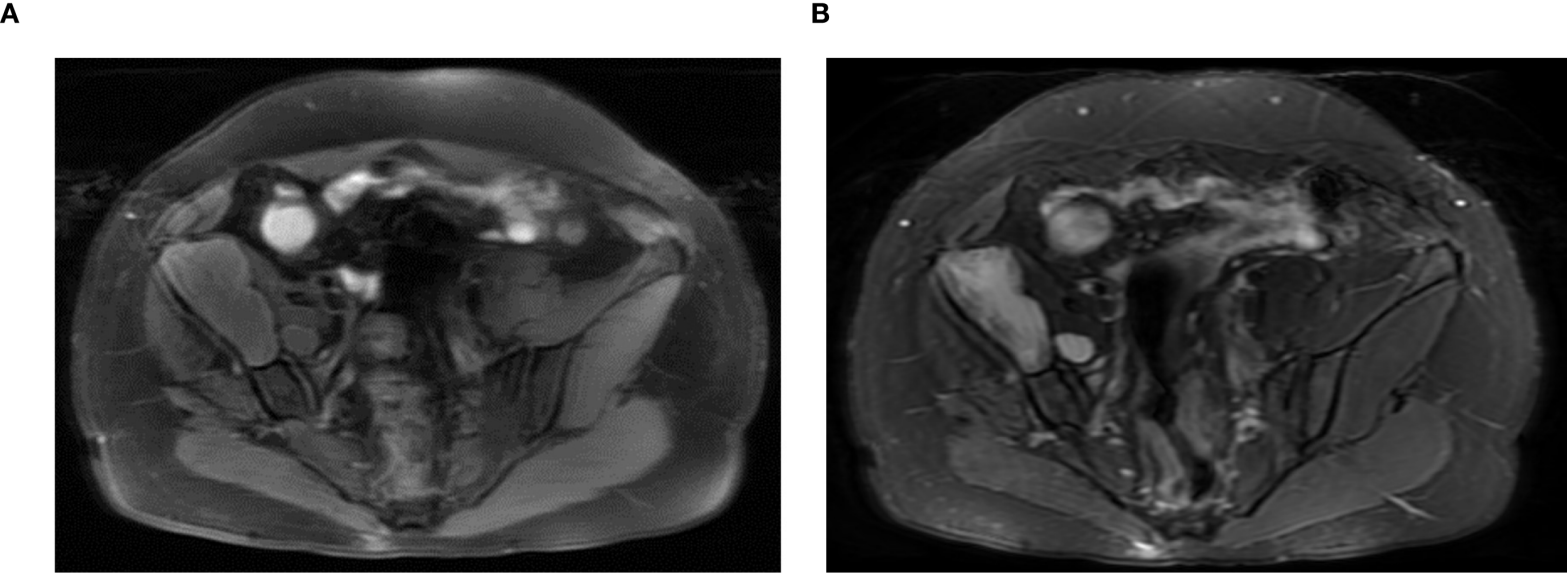
Magnetic resonance imaging (MRI) revealed an iliac mass showing hypo intensity on the T1-weighted **(A)** and hyper intensity on the T2-weighted images **(B)**.

Laboratory examinations revealed no significant abnormalities. The pelvic plain film showed that the joint space of the right hip joint was narrowed, the femoral neck was shortened, and there was ischemic necrosis of the femoral head. Radiographs revealed a large expansile radiolucent lesion, with cortical erosion of the right iliac bone (**Figure 1**). Computed tomography (CT) revealed significant osteolytic destruction in the right iliac wing, along with a mass in the surrounding tissue. Irregular cystic degeneration areas were observed within it. The mass, measuring approximately 10.3 cm × 6.2 cm × 7.8 cm, had an indistinct boundary with muscle tissue (**Figure 2**). Magnetic resonance imaging (MRI) revealed an iliac mass showing hypo intensity.

on the T1-weighted and hyper intensity on the T2-weighted images (**Figure 3**). Diffusion-weighted imaging and apparent diffusion coefficient map showed diffusion restriction of the lesion. MRI findings of diffusion restriction without any enhancement favored the diagnosis of an epidermoid cyst. In view of the progressive trend of the symptoms, the relatively large volume of the mass, the ineffectiveness of conservative treatment, and the quite convincing results of imaging examinations, we recommended giving priority to the surgical removal of the mass at that time, and then performing hip replacement surgery at an appropriate time later. The patient agreed to the surgical intervention and consented to the use of her own imaging and pathological studies for educational purposes.

**Figure 4:**
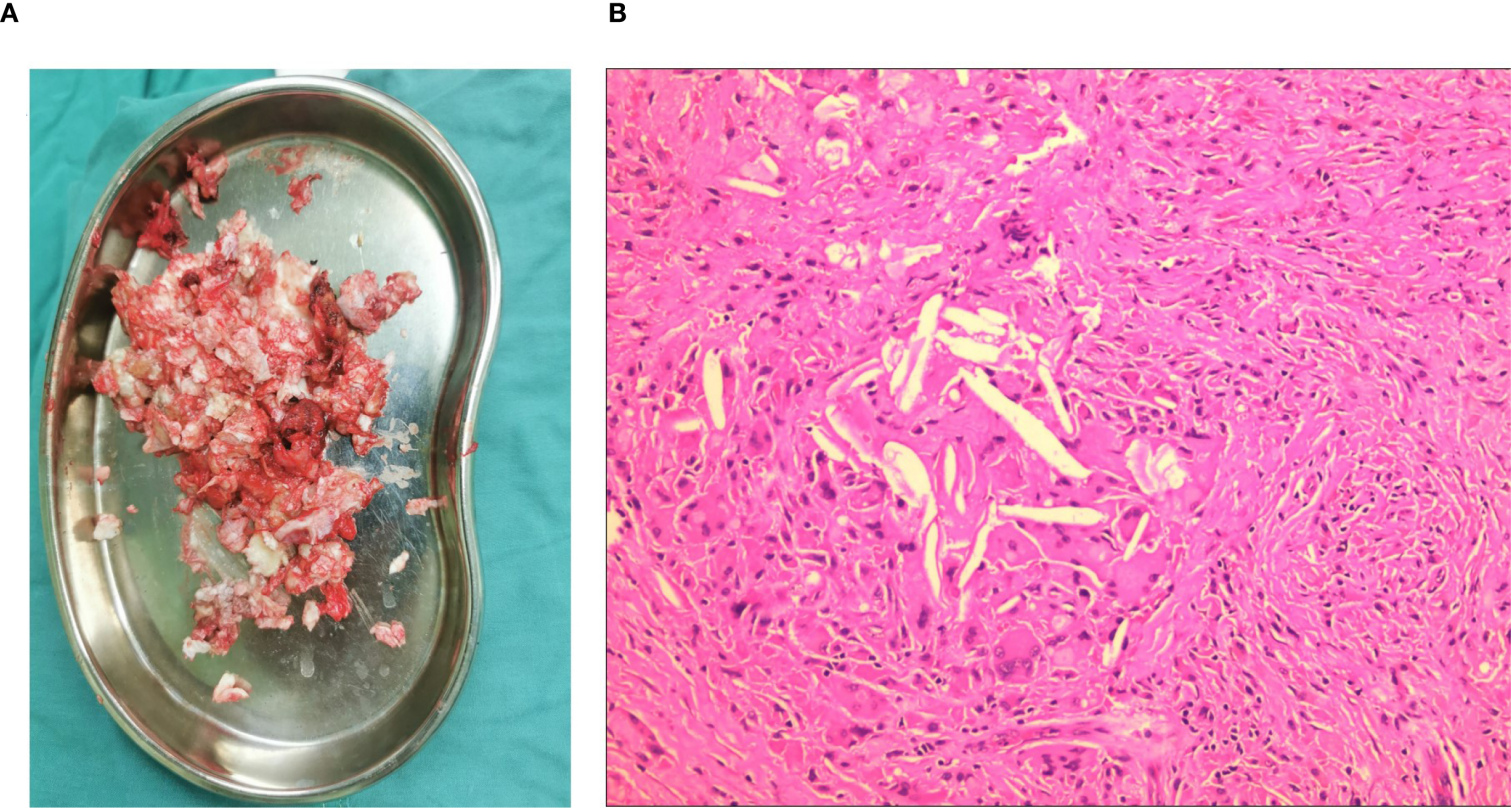
Intraoperative removal of the tumor and pathological examination. The cyst in the ilium was filled with white caseous-like material **(A)**. Micrograph showed the layered squamous epithelium surrounded by a layer of irregular keratinic material (hematoxylin-eosin staining), consistent with the features of an epidermoid cyst **(B)**.

**Figure 5:**
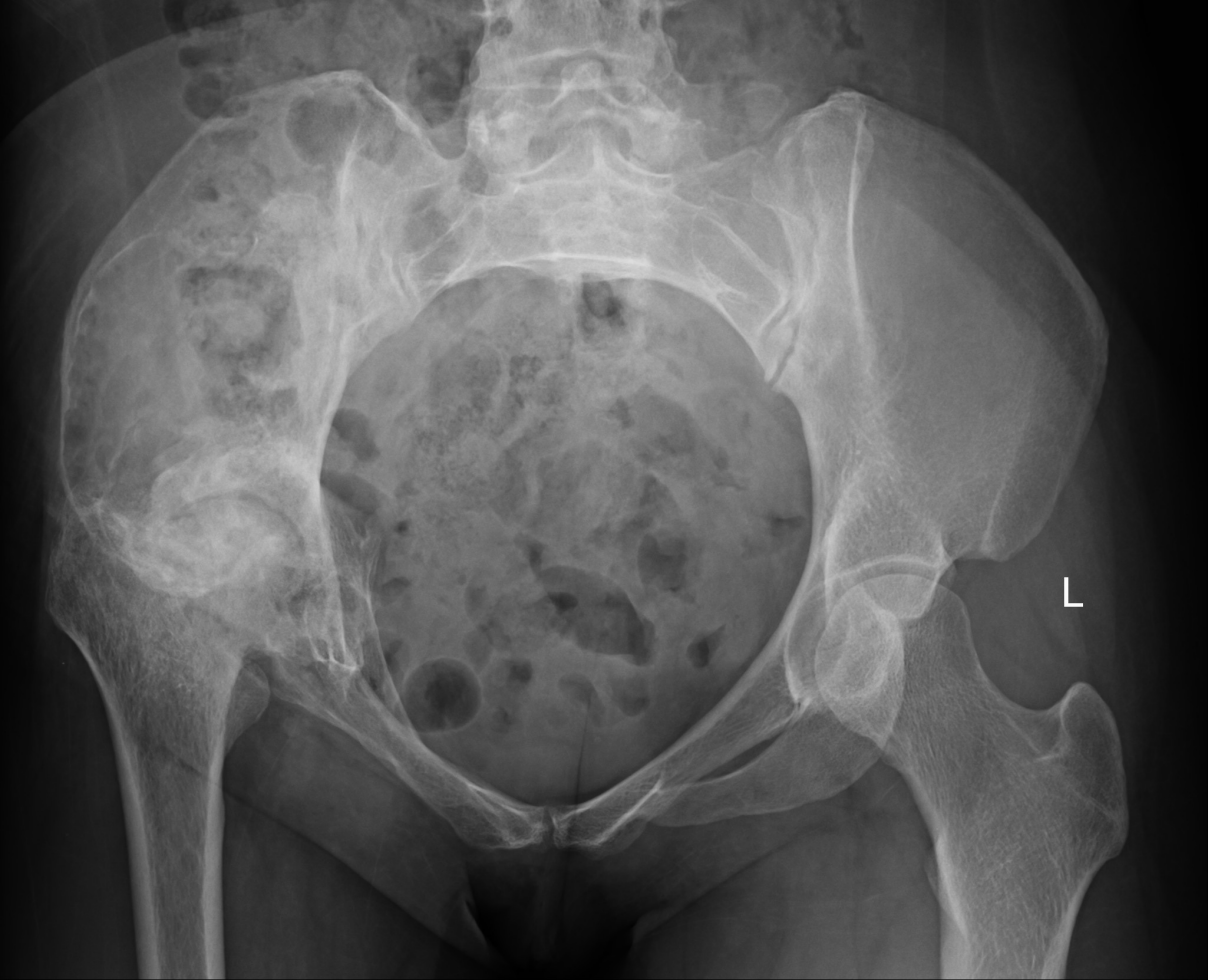
An anteroposterior pelvic radiograph on postoperative day 3 demonstrated no aberrant signal in the right ilium, with partial visualization of the bone graft shadow.

**Figure 6:**
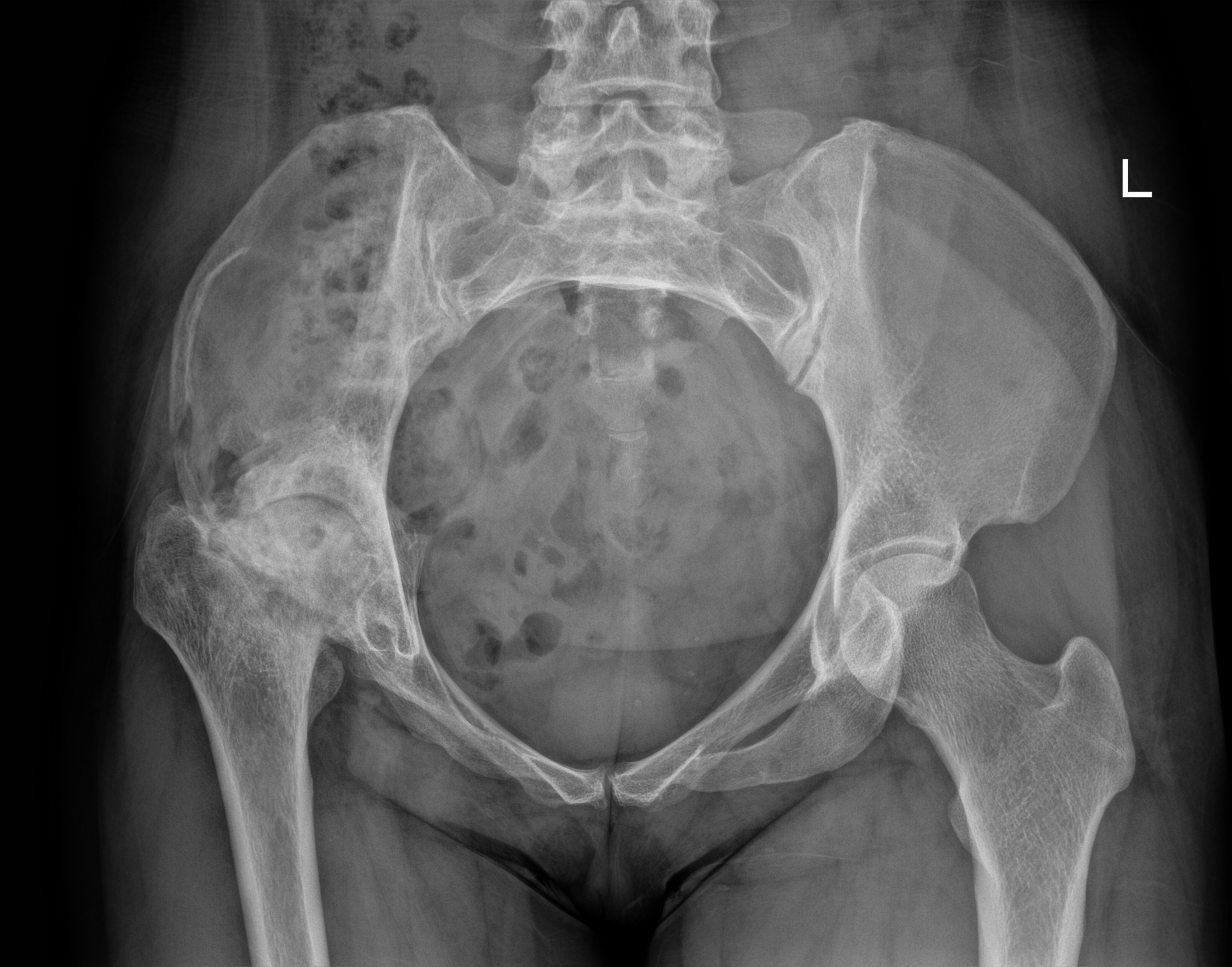
One-year postoperative anteroposterior pelvic radiograph showed good recovery of the right ilium, consistent with postoperative changes.

Enchondroma, giant cell tumors, aneurysmal bone cysts, simple bone cyst, chronic infections and metastatic cancer were considered in the differential diagnosis before surgical exploration. Prior to the operation, a trucut biopsy was conducted, with the result indicated an IEC. An operation was performed to relieve pain and for the histologic diagnosis. Curettage of the lesion and allogeneic bone grafting procedures were carried out. We discovered a certain amount of whitish, caseous, granulation tissue-like material inside the lesion (**Figure 4A**). Postoperative histopathological examination of the lesion showed a dense fibrous connective tissue wall lined by simple stratified squamous keratinized epithelium, and the diagnosis was consistent with an IEC (**Figure 4B**). An anteroposterior pelvic radiograph on postoperative day 3 demonstrated no aberrant signal in the right ilium, with partial visualization of the bone graft shadow (**Figure 5**). The patient was discharged without complications. One year later, the anteroposterior pelvic radiograph showed good recovery of the right ilium, consistent with the postoperative change of the right ilium (**Figure 6**). During the routine follow-up evaluations for one year, she achieved partial alleviation of hip pain. However, substantial functional impairment persisted, manifesting as a pronounced limping gait that significantly impaired ambulation and activities of daily living. The patient will undergo long-term surveillance, with elective total hip arthroplasty scheduled upon clinical requirement.

## Discussion

Intraosseous epidermoid cysts, which are caused by the proliferation of epidermal cells within bones, are rather rare. IEC is a non-neoplastic lesion characterised histologically by a membrane consisting of squamous epithelium, covered by laminated masses of keratin that may occupy part of the cavity (9). They are usually slow-growing and can remain asymptomatic for a long time. They commonly present with swelling, pain, and tenderness at the affected site, and predominantly affect patients between 25 and 50 years of age (10).

Many theories have been proposed regarding the pathogenesis of intraosseous epidermoid cysts which include congenital, traumatic, and iatrogenic etiologies. A traumatic theory remains the most prevalent hypothesis, as many patients have a history of a traumatic event to the involved anatomy prior to symptom onset. The interval between the trauma and the appearance of symptoms varied from one to several years.

Most patients with IECs in the phalanges have a history of blunt or penetrating trauma, suggesting that dermal squamous cells may be implanted into the deep tissues (including bone), and then may develop into an epidermoid cyst (3, 11–14). In addition, congenital IECs are commonly seen in the skull. The cysts have a congenital or developmental origin because most patients with this type of lesion in the skull had no history of blunt or penetrating trauma. The pathomecanism of congenital EC is thought to be the inclusion of ectodermal cells at the time of closure of the neural tube, between the 3rd and 4th weeks of embryonic life (15–17). However, some patients with an IEC in the phalanges had no history of trauma (18), and a few patients with cysts in the skull had a history of trauma (19). Therefore, the precise etiology of IECs is still unclear.

Iatrogenic epidermoid tumours are extremely rare, and although seeding of epidermal cells has been classically described only after lumbar puncture, the same mechanism may be involved after periosteum puncture of the skull (20), surgeries on the skull and lumbar spine (21, 22). Our patient had a well-documented history of undergoing surgical treatment for hip joint tuberculosis 35 years ago. After the operation, the patient’s hip joint movement was significantly restricted, although no other notable discomforts were reported. Although the previous imaging results and surgical records were lacking, the possibility of a pre-existing tumor could be excluded. It is important to highlight that the patient had neither congenital deformities nor skin erythema usually associated with congenital epidermoid cells. It was not until 35 years after the hip surgery that she began to experience progressively worsening pain in the right hip. Imaging examinations suggested hip osteoarthritis, avascular necrosis of the femoral head, and a substantial mass on the ipsilateral ilium. Preoperative puncture biopsy of the mass confirmed it as an epidermoid cyst of the ilium. Considering the patient’s previous history of hip surgery and the perfect match between the location of the primary surgical site and the subsequently occurring epidermoid tumor, we strongly propose that the IEC in this case was formed after the hip surgery.

This neoplasm might have arisen from the inadvertent implantation of epidermal cells into the ilium during the prior surgical intervention for hip joint tuberculosis. These dislocated cellular tissues then grow slowly and it is only when the mass is sufficiently large to compress the adjacent tissues that the patient will have the related clinical manifestations.

Radiographically, IEC typically presents as a well-defined osteolytic lesion without any trabecular pattern. The cortex is expanded and thinned, but bony sclerosis or reaction is typically absent (3). It was difficult to distinguish the nature of the lesion using radiographs alone. Enchondroma, giant cell tumors, aneurysmal bone cysts, simple bone cyst, chronic infections and metastatic cancer are other lesions that must be differentiated (1, 5, 23). IEC has specific imaging findings, especially in MRI that can help in the diagnosis. The lesions follow fluid-like signals in T1 and T2 weighted images. however, focal areas of hypointensity can be seen, reflecting the presence of dependent debris. There may be rim enhancement of the surrounding cyst wall following gadolinium administration. The enhancement around the cyst is due to fibrosis and giant-cell reaction to keratin (24). This imaging evidence lacks the specificity required for the definitive diagnosis of an IEC. Therefore, a histopathological test of the lesion tissue is the final resort to confirm the diagnosis of intraosseous epidermoid cysts among the various differential diagnoses. In our case, for a definite diagnosis, we performed a percutaneous biopsy before the operation. The histopathological analysis presented that the lesional tissue contained keratinous cell debris surrounded by a wall of the stratified squamous epithelium, which confirmed the diagnosis of IEC.

Most IECs are harmless. Rarely, a benign lesion might transform into a malignant lesion (25). The surgical treatment of IEC is intraregional curettage. To prevent the recurrence of IECs, complete curettage that includes the wall of the cyst and en bloc excision of the lesion with the surrounding soft tissue is needed. Whether bone grafting is requisite remains a subject of debate. In the absence of cortical thinning, the necessity for bone grafting may be obviated (3). Given that our patient had a relatively large area of bone defect and the lesion had already involved the acetabulum, in order to prevent the occurrence of pathological fractures, we carried out allogeneic bone transplantation. Meanwhile, this also prepared sufficient bone mass for the second-stage hip replacement.

In conclusion, we believe that this is the first reported case in the literature of an acquired IEC secondary to surgery for hip joint tuberculosis. Despite the extreme rarity of this event, we should be aware of the potential *de novo* development of epidermoid cyst in patients who underwent surgery in the pelvic region, especially those related to surgery for hip joint tuberculosis. Furthermore, its clinical and imaging manifestations are highly similar to those of other tumors, and only through histopathological examination can a definite diagnosis be made. The most effective treatment of IEC is Intra-lesional curettage with or without bone graft. In the case of large-scale bone defects, bone grafting might be requisite. A complete removal of the cyst results in a low recurrence rate.

## Data Availability

The original contributions presented in the study are included in the article/**Supplementary Material**. Further inquiries can be directed to the corresponding author.
